# Outbreak of occupational *Brucella* infection caused by live attenuated *Brucella* vaccine in a biological products company in Chongqing, China, 2020

**DOI:** 10.1080/22221751.2022.2130099

**Published:** 2022-10-26

**Authors:** Chunbei Zhou, Wenli Huang, Xu Xiang, Jianping Qiu, Dayong Xiao, Ning Yao, Qiang Shu, Shuang Zhou

**Affiliations:** a Chongqing Center for Disease Control and Prevention, Chongqing, People’s Republic of China; b Rongchang District Center for Disease Control and Prevention, Chongqing, People’s Republic of China; c Army Medical University (Third Military Medical University), Chongqing, People’s Republic of China

**Keywords:** *Brucella*, brucellosis, outbreak, case-control study, vaccine

## Abstract

The northern areas of China are traditional endemic regions for brucellosis in both animals and humans, while occasional outbreaks of brucellosis have been observed in neglected southern provinces. On 16 December 2020, Chongqing Center for Disease Control and Prevention (CQCDC) received a report of 15 *Brucella* seropositive employees in a biological products company. The CQCDC and the local health administrative department launched an investigation that included identification of cases, laboratory testing of samples, and employees’ interview to identify the cause of this incident. A case-control study was implemented to compare high-risk factors between cases and serology-negative personals. Human and animal serum samples and environmental swabs were collected for testing. A total of 61 recessive infectors were found with an infection rate of 43.57% (61/140). Fisher’s exact test showed that there were significant differences in *Brucella* infection rates among different post classifications (*p *= 0.02), working places (*p *= 0.007), and buildings (*p* < 0.0001). Case-control study showed that working in vaccine production workshop was independently associated with an increased risk of infection (odds ratio (*OR*): 2.60; 95% confidence interval (*CI*): 1.31–5.19). The positive detection rate was 88.06% (59/67) for production environment and 16.67% (2/12) for external environment. The investigation indicated that close contact with biological products and aerosol were the potential transmission routes of this outbreak under the condition of insufficient personal protection and disinfection. Our study provides new epidemiological evidence for a more detailed understanding of occupational infections with live attenuated *Brucella* vaccine.

## Highlights

- An Outbreak of occupational *Brucella* infection was caused by *Brucella* vaccine.
- Contacting with biological products and aerosol were potential transmission routes.
- This is new evidence for the virulent characteristics of attenuated *Brucella* vaccine.

## Introduction

Brucellosis is a worldwide zoonotic infectious disease caused by *Brucella* bacteria, and remains a considerable public health problem in many low-income and developing countries [1,2]. The most common clinical symptoms and signs of human brucellosis are prolonged fever, fatigue, sweats, chills, body aches, arthralgia, weight loss, and weakness [3,4]. Human brucellosis can be transmitted from infected livestock to humans via inhalation or ingestion of bacteria, conjunctiva, or skin abrasions [5], and is also associated with the consumption of unpasteurized milk from infected livestock or close exposure to their secretions [6]. Although the transmission of brucellosis from human to human has been reported in the literature [7], this condition is rare.

Over 500,000 human cases of brucellosis are reported worldwide every year [8], and brucellosis is also found to expand in animals and humans in the Pacific region and Asian countries, including China [9–11]. It is reported that as a class B infectious disease, human brucellosis has been endemic in more than 25 of 32 provinces or autonomous regions in mainland China [6], and a total of 448,000 cases were detected in China during 2004–2016 [12]. The northern areas of China are found to be traditional endemic regions for brucellosis in both animals and humans by the National Notifiable Disease Surveillance System (NNDSS) [13], while outbreaks of brucellosis have also been observed in neglected southern provinces. In 2018, an outbreak of brucellosis via air-born transmission in a kitchen wastes disposing company in Lianyungang, China, was reported with an attack rate of 33.3% [14]. Zhan BD et al. reported an occupational outbreak of brucellosis infection in a pharmaceutical factory in Southeast China in 2015 [6]. Previous studies have shown that people with occupational exposure were at higher risk for human brucellosis, especially for workers and farmers from butcheries, trading stockyards, and markets in southern China [15]. Therefore, human brucellosis is also considered an occupational disease because it typically occurs in occupational activities where workers are exposed to *Brucella*, such as animal husbandry, meat processing, and vaccine production industries [16].

In this study, we investigated an outbreak of occupational *Brucella* infection in a biological products company in Chongqing, in Southwest China. Field epidemiological investigation, laboratory tests, case-control study, and targeted hierarchical strategies of exposure/control were conducted to identify the cause of the outbreak. It can provide scientific basis for supervision and administration of this event and help for preventing occupational outbreaks of human brucellosis in future.

## Material and methods

### Case report

On 16 December 2020, Chongqing Center for Disease Control and Prevention (CQCDC) received a report of *Brucella* infection in a biological products company in Chongqing, and 15 persons (15/23) were tested serologically positive for *Brucella* during the occupational health check-ups. The company is mainly engaged in the production of veterinary vaccines, and the live *Brucella* vaccines are attenuated strain *Brucella* abortus A19 and S2.

### Descriptive epidemiological investigation

According to the Chinese diagnostic criteria for brucellosis (WS269-2019), a suspected case was defined as any worker of the biological products company who developed two or more of the following symptoms: prolonged fever, sweating, fatigue, and arthralgia excluding patients with confirmed diagnosis for other diseases. A confirmed case was defined as suspected case with positive 1:100 (++) or above serum agglutination test (SAT) for *Brucella*. A recessive infector was defined as any person whose SAT result was positive without any symptoms. Active case searching was conducted among all workers and managers in the company. We also searched local hospital records and outpatient clinical files, reviewed Infectious Disease Reporting Management Information System in order to find additional cases. Each case was interviewed face to face by trained public health officers after verbal consent. Information about demographic characteristics, clinical symptoms, date of onset, occupational history, working situation, preventive measures, and source of animal products was collected through a structured questionnaire.

### Analytical epidemiology

Hypothesis was generated based on the time, place, distribution of cases and the apparent infection history in this company, exposure to *Brucella* at a production stage might be the source of infection. In order to test this hypothesis, we conducted a case-control study by interviewing all employees of the company with informed consent. All patients including confirmed cases and recessive infectors were selected as cases and all remaining employees were included in control group.

### Environmental hygiene investigation

We inspected the disinfection facilities and environmental sanitation in the production workshop, products research and development centre, restaurant, and public activity areas of the company. A further investigation was performed, including the production process of vaccines, the availability of protective equipment, air-conditioning, and sewage system.

### Specimen collection and laboratory test

Serum samples of all employees in the biological products company were collected and tested in CQCDC laboratory according to Chinese diagnostic criteria for brucellosis (WS269-2019). Blood samples from 19 cases were cultured for *Brucella*. Environmental samples were collected from possible polluted sites of the company, including production workshop, quality inspection centre, warehouse, and public area. Meanwhile, serum samples of susceptible animals for inspection were collected. Animal and environmental samples were sent to the National Reference Laboratory for animal brucellosis (ANSES), rose bengal plate agglutination test (RBT), and real-time PCR (RT–PCR) were then performed.

### Statistical analysis

Data were entered in an Excel spreadsheet and all analysis was performed with R, version 3.1.2 (R Foundation). The distribution of cases was summarized based on the frequencies and percentages. The “chisq.test” and “fisher.test” functions of the Stats package in R were used to compare the characteristics and exposure of cases and controls. The Chi-square trend test and bivariate analyses were performed on hypothesized possible polluted environments which these cases were exposed to. The odds ratio (*OR*) and the corresponding 95% confidence interval (*CI*) were reported. All statistical tests were two-tailed and *p*-value < 0.05 was considered significant.

## Results

### Descriptive epidemiology

At the time of this incident, there were 140 employees in the biological products company. They were all sampled during the case search and 61 recessive infectors were found with an infection rate of 43.57% (61/140). All cases were tested seropositive for *Brucella* but with no clinical signs, no suspected case or confirmed case was found. No *Brucella* bacteria were cultured from blood samples collected from the 19 recessive infectors. Among the 61 cases, 30 were male and 31 were female, with no significant difference in infection rate between genders (*χ^2 ^*= 0.38, *p *= 0.54). The median age of the cases was 35.6 years old (range: 21–57). Fisher’s exact test showed that the infection rate of *Brucella* was significantly different among different posts classifications (*p *= 0.02) and working places (*p *= 0.007). The infection rate of *Brucella* was highest in building E (66.67%), following by building C (55.84%), and then building A (38.10%) (Table 1).

**Table 1.** Infection rate across different demographics in the outbreak of occupational *Brucella* infection occurred in the biological products company, Chongqing, China, 2020.

Variables	Total	No. of cases	Infection rate (%)	*χ^2^*	*p*
Gender					
Male	73	30	41.10	0.38	0.54
Female	67	31	46.27		
Age (years)					
20—29	27	18	66.67	7.63	0.05
30—39	57	22	38.60		
40—49	40	16	40.00		
50—59	16	5	31.25		
Length of service (years)					
<1	14	5	35.71	1.82	0.61
1–2	24	13	54.17		
3–4	49	22	44.90		
≥5	53	21	39.62		
Posts classification					
Vaccine production	73	41	56.16	–	0.02*
Laboratory quality control	10	4	40.00		
Experimental preparation	11	4	36.36		
Logistician	42	12	28.57		
Animal caretaker	4	0	0		
Work place					
Vaccine production workshop	71	39	54.93	–	0.007**
Vaccine sub-packaging workshop	65	22	33.85		
Animal laboratory	4	0	0		
Building					
A	21	8	38.10	-	<0.0001**
B	29	4	13.79		
C	77	43	55.84		
D	4	0	0		
E	9	6	66.67		

Note: – Fisher’s exact test. *p < 0.05, **p < 0.01.

**Figure 1.**
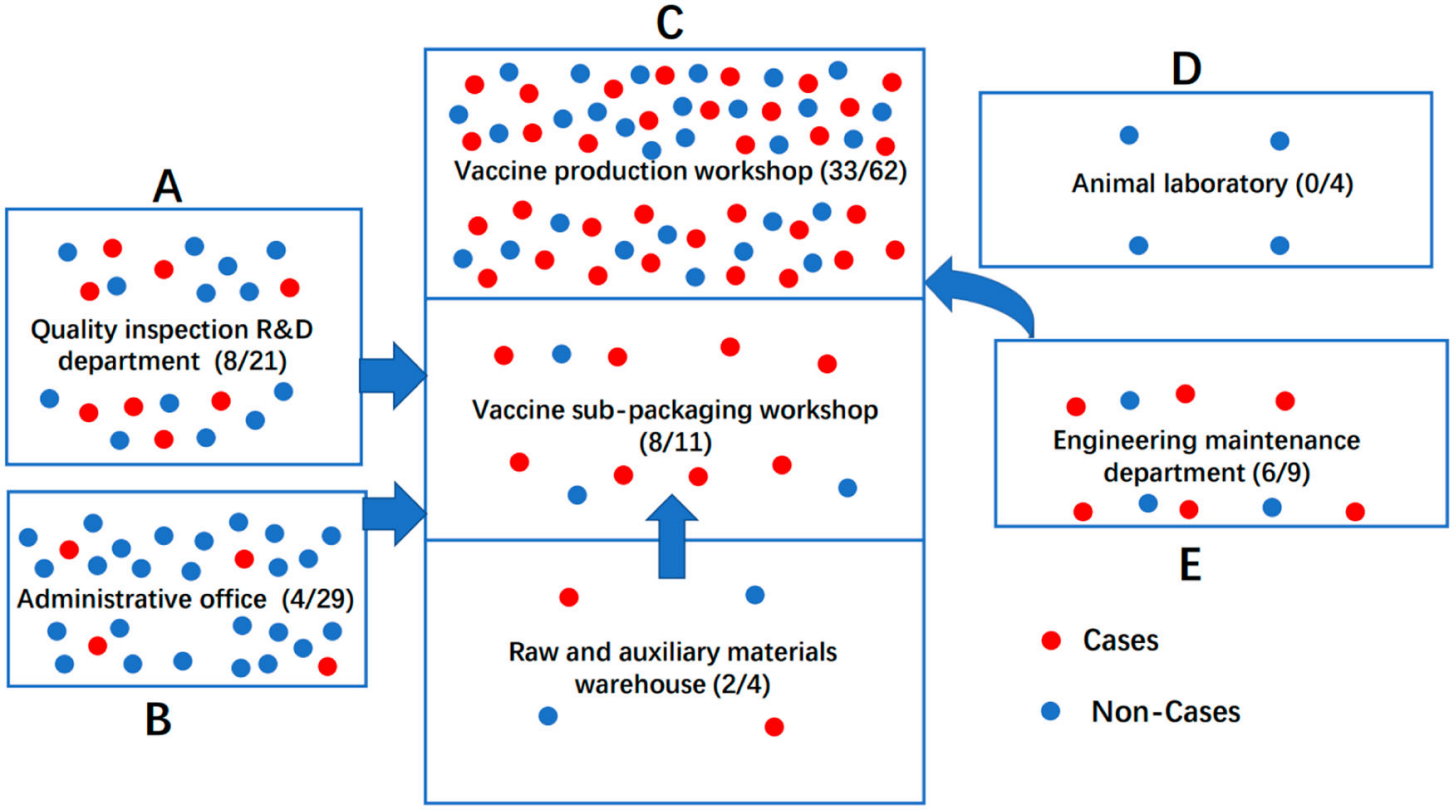
Spatial distribution of employees in the outbreak of occupational *Brucella* infection occurred in the biological products company, Chongqing, China, 2020.

Further investigation found that all employees had the same traditional Chinese buffet lunch provided by the company canteen every day. The company’s tap water is from the local water plant. Vaccine production is seasonal in this company and during the production peak in spring and autumn, the company will flexibly allocate logistical employees to work on the production line, including managers, electricians, cleaners, and drivers. In late autumn, November 2020, the staffs in administrative office and other logistical departments were asked to help packaging vaccines in the packaging workshop, and the workers of the engineering maintenance department were regularly arranged to repair the equipment in the vaccine production workshop. Only 4 employees of the animal laboratory have never participated in the above work (Figure 1).

Based on the above results, the vaccine production workshop and vaccine sub-packaging workshop were the most likely sites of *Brucella* exposure for workers. Significant difference in *Brucella*-infected rates between workplaces was observed, and the Cochran–Armitage trend test was significant (*χ^2 ^*= 9.05, *p *= 0.003) (Table 2).

**Table 2.** Infection risk among different workplaces in the outbreak of occupational *Brucella* infection occurred in the biological products company, Chongqing, China, 2020.

Possible exposure place	Cases	Controls	*OR*
Animal laboratory (Ref)	0	4	1.00
Vaccine sub-packaging workshop	22	43	+∞
Vaccine production workshop	39	32	+∞

**Figure 2.**
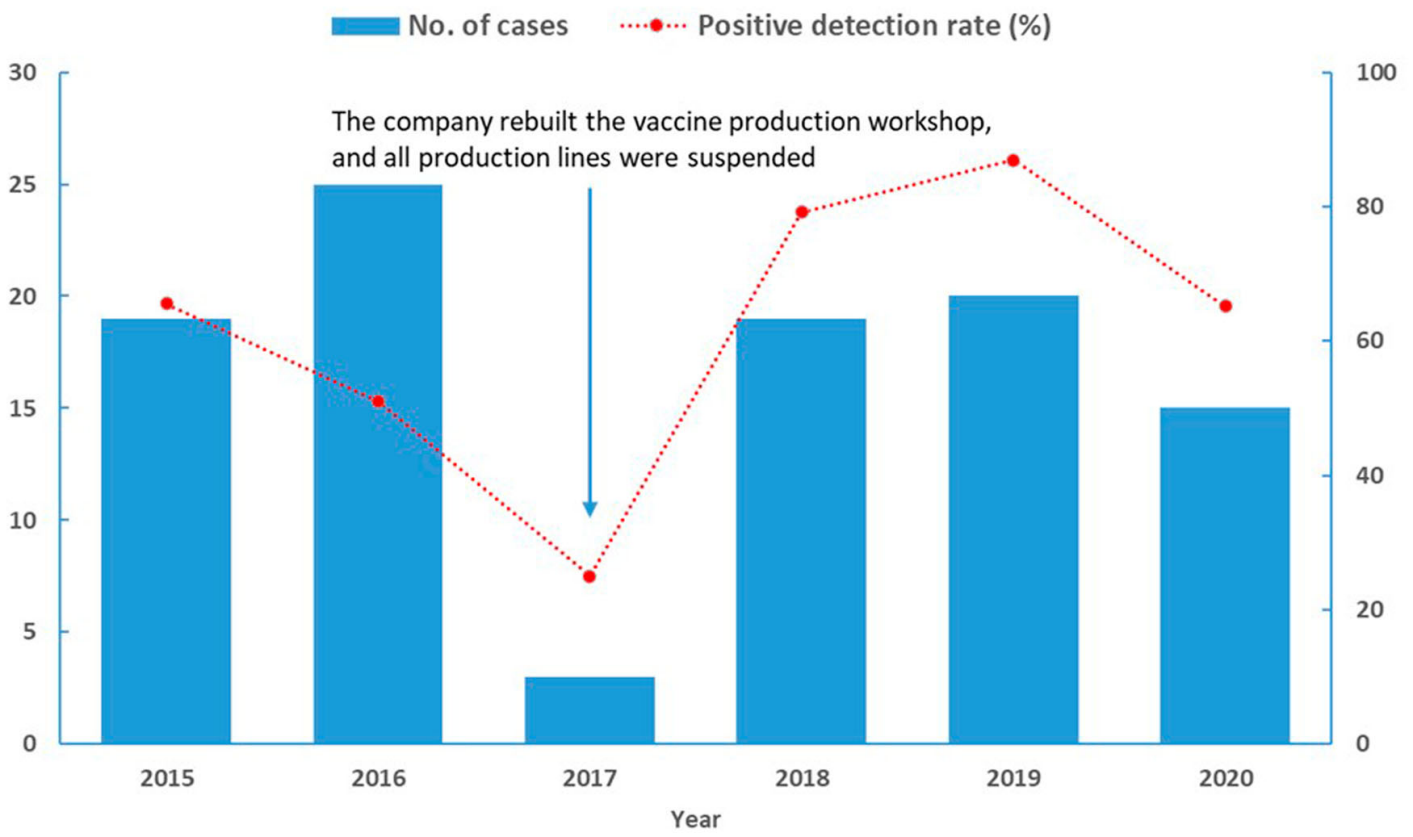
The employees in the vaccine production workshop of the company detected positive for *Brucella* through occupational health examinations, 2015–2020.

In the retrospective review, the company obtained the approval for the production of veterinary *Brucella* vaccines in 2014, and started to produce vaccines since 2015. Some employees who have high-risk of *Brucella* infection were sampled and received occupational health examinations every year from 2015 to 2020. A total of 160 person-times received *Brucella* serological tests, of which 101 person-times were serologically positive, accounting for 63.13% (Figure 2). In this general investigation, 5 of the 61 cases were previously infected.

### Analytical epidemiology

A total of 61 cases and 79 controls were involved in the case-control study. Working in vaccine production workshop (*OR*: 2.60, 95%*CI*: 1.31–5.19) was associated with the raised risk of *Brucella* infection (Table 3).

**Table 3.** Crude odds ratios for statistically significant association between *Brucella* infection and working places in the biological products company, Chongqing, China, 2020.

Working place	Exposures		%		*OR* (95%*CI*)
	cases (*n* = 61)	Controls (*n* = 79)	Cases	Controls	
Vaccine production workshop	39	32	63.93	40.51	2.60 (1.31–5.19)
Vaccine sub-packaging workshop	22	43	36.07	54.43	0.47 (0.24–0.94)

### Environmental hygiene investigation

**Figure 3.**
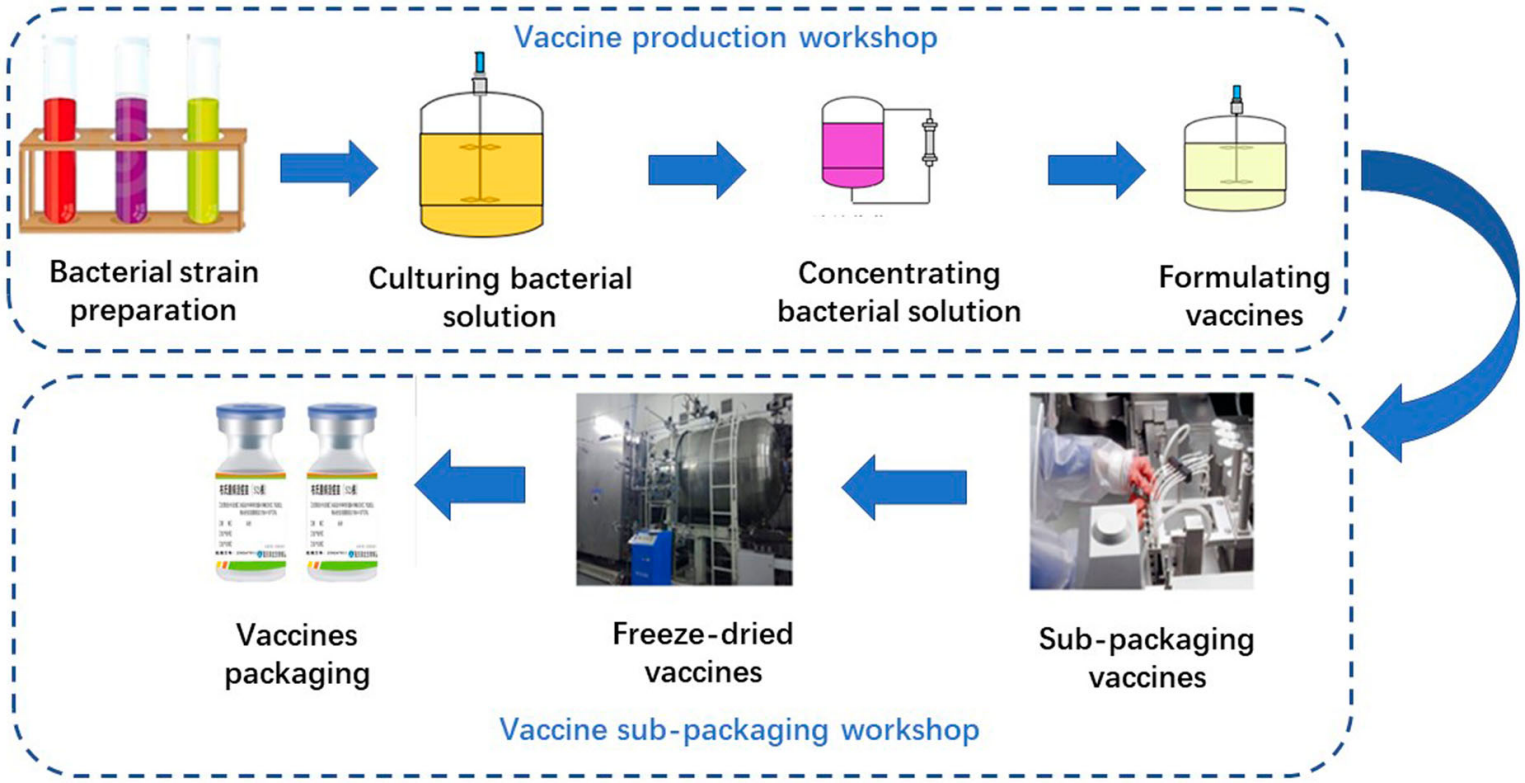
The main production process of live attenuated *Brucella* vaccine in the biological products company, Chongqing, China.

The survey showed that the production process of live attenuated *Brucella* vaccine mainly contains 7 steps (Figure 3), and the possible exposures included: (1) The unsealing of bacteria and the preparation process were performed manually in open conditions. (2) In the process of bacterial solution filling, although the air pressure in the sub-packaging room was negative, the operation was performed under the partly 100-level positive pressure conditions without using protective covers, which may lead to dispersion of *Brucella*-containing aerosols. (3) During the production of live *Brucella* vaccine, although the two sets of independent air-conditioning systems were used in the *Brucella* vaccine cultivation and filling area, workers entering and exiting through one channel would cause air cross contamination. (4) As orders increased, some workers without professional training temporarily participated in the vaccine packaging work. (5) The effectiveness of the ozone disinfection method in the packaging room had not been verified, and the surface disinfection of the vaccine bottle was possibly not thorough enough, which would absorb *Brucella* in the aerosol. (6) In the vaccine production workshop, individual workers took off the goggles for a short time after the goggles fogged during the operation, and several workers were even found not wearing masks and gloves when sub-packaging vaccines.

Since the wastewater produced by the company has been sterilized by steam and then discharged into the sewage treatment station, and the gas has also been efficiently filtered before being discharged to the outside environment, the risk of being infected by *Brucella* for the surrounding population was relatively low.

A total of 35 animal serum samples were collected, including 30 rabbits and 5 guinea pigs, and all of them were negative by RBT. Seventy-nine environmental samples were taken from 27 different locations of the company, including 67 internal environmental samples and 12 external environmental samples. RT–PCR results showed that the positive detection rate of samples from areas related to *Brucella* vaccines production was 88.06% (59/67), and the positive detection rate of samples outside the production areas was 16.67% (2/12) (Table S1).

### Emergency response to this outbreak

All 140 employees were organized to receive health examinations in the local comprehensive hospital, and those who tested positive for *Brucella* were further examined for related symptoms and signs. The production of vaccines was stopped immediately and all places were fully disinfected (including the production workshop and the external environment). Extra measures were implemented including strengthening the production safety training, provision of personal protective equipment (PPE), and standardization of safety operation procedures. The industry experts were organized by local agricultural departments to evaluate the safety of vaccine production and the effects after the proper rectification.

## Discussion

Our investigation confirmed that it was an outbreak of occupational *Brucella* infection among workers in a biological products company in Chongqing, China, in December 2020. Due to the timely detection and decisive control measures of the epidemic, the outbreak did not spread to surrounding areas. Based on the results of epidemiological investigation, laboratory test, and environmental hygiene investigation, we identified that the most probable workplaces for *Brucella* exposure were the production workshop and sub-packaging workshop. The unsealing of bacteria and the preparation process were conducted by workers with no protective covers in the opening environment, which might lead to the release of live *Brucella* vaccine into the space. Person who enters the workplace is at risk of direct exposure to live *Brucella* vaccines. In addition, workers’ poor awareness of protection and inadequate protective measures has increased the risk of touching/inhalation of live *Brucella* vaccines.

Occupational infection of brucellosis is common in China, but it is rare for so many *Brucella* infected found in an outbreak in a southern Chinese city. In recent years, the burden of brucellosis infection in China has gradually spread from northern pastoral and semi-pastoral areas to southern non-pastoral areas [17,18]. One of the important reasons is that with the increased livestock trading between the north and south, and the increased private free-range livestock in the south of China, many brucellosis infections in southern provinces result from imported infected livestock or products from Northern China [6,19]. An outbreak of human brucellosis in Jiangsu Province was caused by the plentiful influx of unchecked sheep from Northern China and the lack of an effective prevention programme [20]. Zhang Z et al. reported the first outbreak of occupational brucellosis involving multiple clusters in Hubei Province, China in 2019, which was due to the influx of infected goats and mutton [21]. Importing an infected goat was the core link that may lead to the local brucellosis outbreak in Fujian Province, China [22]. Reports of human brucellosis caused by occupational exposure in farm, slaughter, kitchen wastes disposing company, and even microbiologic laboratory have been documented in previous studies [14,23–25], while the outbreak of brucellosis in the biological products company was rarely reported.

Some scholars have reported an occupational outbreak of brucellosis infection in a pharmaceutical factory in Southeast China, and exposure was after close contacting with sheep placentas without thorough disinfection [6]. Similarly, Yoshida GJ reported two outbreaks of brucellosis between 2013 and 2015 in the pharmaceutical manufacturing industry, and the confirmed source of infection was sheep placenta [26]. The common exposures to *Brucella* found in the above reports were all due to insufficient personal protection and disinfection after close contact with biological products. Similar problems also existed in this outbreak of Chongqing. However, the difference was that the former was exposed to dead animals, while the latter was exposed to live vaccines. Our investigation found several risk factors of transmission of *Brucella* in production. Some steps of vaccine production were performed in open conditions without using protective covers, the effectiveness of the ozone disinfection method in the packaging room had not been verified, and individual workers took off goggles or did not wear masks and gloves during the process of sub-packaging. All the above would possibly increase the risk of infecting *Brucella* for workers. It is known that the production of veterinary vaccines has strict operating procedures. Unsealing of bacteria and the filling of bacterial solution are performed manually in an open environment, which poses a potential risk for exposure to *Brucella*. The lyophilized vaccines are easy to form aerosols and spread rapidly in the closed space if the operation is improper, which could significantly increase the number of infected individuals. Therefore, standardized wearing of PPE and adequate disinfection after potential exposure are particularly important. In addition, many workers without professional training temporarily participated in vaccine production and subcontracting work is also contributed to the large number of persons infected with *Brucella* in this outbreak.

In 2008, an Argentine study of infection by *Brucella* abortus S19 among workers from vaccine-manufacturing plants found that active brucellosis was diagnosed in 21 persons of 30 employees. The positive rate (70.00%) was significantly higher than that of the outbreak in Chongqing (43.57%) [27]. The live *Brucella* vaccines produced by the biological product company in Chongqing are attenuated strain *Brucella* abortus A19 and S2. The A19 strain is derived from the S19 strain, which was a very stable attenuated vaccine developed in the 1920s. It has a good immune effect on cattle and is widely used in many countries. The S2 strain is currently the least virulent strain known in the world and is the most widely used to prevent porcine brucellosis, with high genetic stability. The virulence of these two vaccines is very stable and weak, and they are used for the production of live attenuated *Brucella* vaccines for animals that can be used safely, which may explain why all the positive infected persons did not have any clinical symptoms in this outbreak. The antibodies caused by a small dose of *Brucella* vaccine will decay after entering the human body in 3–6 months. A study performed in Jilin Province, China, has found that asymptomatic infection patients had lower antibody titres, which might result from lower frequency or longer duration of infection [28]. Contrary to our findings, other studies have demonstrated that *Brucella* vaccine infection can cause overt clinical disease in humans [27,29]. In a detailed study about the characteristics of physiological and attenuated virulence of the A19 strain, the virulence test showed that the A19 was significantly attenuated at the chronic infection stage in infected mouse model, and the A19 was not cytotoxic for macrophages, reduced its ability to invade, survive, and traffic within host cells [30]. Our study provides new epidemiological evidence for the virulent characteristics of attenuated *Brucella* vaccine and will be beneficial for the improvement of current *Brucella* vaccine.

Chongqing is located in the southwest of China. Although it is not a traditional pastoral area, the infection of brucellosis in humans and animals has been on the rise since the first local case of human brucellosis was reported in 2011. The clinical symptoms of human brucellosis are atypical, and cases are easily misdiagnosed and overlooked by doctors in primary hospitals [31–33]. Therefore, in non-endemic areas, it is paramount to prevent the import of infected animals, enhance recognition and knowledge of brucellosis in doctors, occupational workers, and the general public for effective control of human brucellosis [34–36].

Although there were no symptoms after infection with live attenuated *Brucella* vaccines of animals, positive cases have been consistently identified over several years, indicating the risk of infection may be existed persistently for years and has not been well controlled. Targeted measures against occupational infection of human brucellosis should be considered throughout the production process. Optimizing production processes, improving general ventilation with air changes, and ensuring the normal use of disinfection equipment can greatly reduce the probability of exposure to *Brucella*. Biological companies should provide effective PPE for workers and urge them to wear it strictly. Health education including occupational health training, PPE management, and good hygiene habits among relevant occupational groups and enterprise managers would be helpful to prevent the occupational outbreak of brucellosis in future. The occupational medical examination, epidemic monitoring of employees, and occupational disease risk assessment should be persistently implemented. In addition, the serologic surveillance of brucellosis in animals and the immunization of herds are also essential preventive measures [36].

There were two limitations in our study. Firstly, test of all human samples was SAT positive, but *Brucella* was not tested in the cultured blood samples of cases. The gold standard diagnostic method is *Brucella* culture in spite of time-consuming and low positive rate [6]. Secondly, based on the epidemiological evidence and environmental hygiene investigation, close contact and aerosol were potential routes of transmission in these cases, we did not have enough evidence to confirm the exact routes of transmission.

## Conclusions

The outbreak of occupational *Brucella* infection in the biological products company in Chongqing, China, was caused by live attenuated *Brucella* vaccines for animals. We speculate that employees were exposed to *Brucella* in the production workshop and sub-packaging workshop, and close contact or aerosol were potential routes of transmission in cases. It is of great importance to reinforce the standardized operation of occupational workers in the production process to mitigate occupational exposure to *Brucella* in biological products companies. Sufficient disinfection and individual protection should be strengthened with annual health examinations and sustained symptoms monitoring to prevent similar occupational infections.

## Supplementary Material

Supplemental Material

## Data Availability

The data presented in this study are available on request from the corresponding author.
