# Supplementary material for: Outbreak of occupational Brucella infection caused by live attenuated Brucella vaccine in a biological products company in Chongqing, China, 2020
Source: Emerg Microbes Infect. 2022 Oct 26;11(1):2544–52. doi: 10.1080/22221751.2022.2130099 (PMC9621275; doi:10.1080/22221751.2022.2130099)
Supplement: Supplemental Material [file TEMI_A_2130099_SM7824.zip › Highlights.docx]

Highlights

• An Outbreak of occupational *Brucella* infection was caused by *Brucella* vaccine.

• Contacting with biological products and aerosol were potential transmission routes.

• This is new evidence for the virulent characteristics of attenuated *Brucella* vaccine.
