# Supplementary material for: Outbreak of occupational Brucella infection caused by live attenuated Brucella vaccine in a biological products company in Chongqing, China, 2020
Source: Emerg Microbes Infect. 2022 Oct 26;11(1):2544–52. doi: 10.1080/22221751.2022.2130099 (PMC9621275; doi:10.1080/22221751.2022.2130099)
Supplement: Supplemental Material [file TEMI_A_2130099_SM7824.zip › Table S1.docx]

**Table S1.**  RT-PCR results of environmental samples.

| **Location** | **Sampling position** | **Sample number** | **Ct value** | **Result** |
| --- | --- | --- | --- | --- |
| Bacteria seed preparation room | Air outlet | 1 | 34.60 | + |
|  | Table | 2 | 30.98 | + |
|  | Ground | 3 | 26.26 | + |
|  | Air inlet | 4 | 25.49 | + |
| Fermentation room | Wall | 5 | 26.25 | + |
|  | Air outlet | 6 | None | - |
|  | Wall | 7 | 34.69 | + |
|  | Fermentation rack | 8 | None | - |
|  | Air outlet | 9 | 31.86 | + |
| Purification and concentration room | Air outlet | 10 | 35.81 | + |
|  | Air outlet | 11 | 29.74 | + |
|  | Wall | 12 | 31.99 | + |
| Test room | Wall | 13 | 9.33 | + |
|  | Air outlet | 14 | 30.25 | + |
|  | Table | 15 | 31.98 | + |
| Greenhouse | Table | 16 | 33.30 | + |
|  | Air outlet | 17 | 29.72 | + |
| Sub-packing workshop | Air outlet | 18 | 27.72 | + |
|  | Table | 19 | 20.55 | + |
|  | Table | 20 | 19.26 | + |
|  | Air outlet | 21 | 31.46 | + |
|  | Ground | 22 | 21.66 | + |
| Capping room | Table | 23 | 23.96 | + |
|  | Air outlet | 24 | 30.41 | + |
|  | Transfer window | 25 | 31.61 | + |
| Disinfection valve room | Ground/Wall | 26 | 23.18 | + |
| Packing room | Table | 27 | 32.68 | + |
|  | Wall | 28 | 28.44 | + |
|  | Air outlet | 29 | 25.81 | + |
| Out-porch | Ground/Wall | 30 | 32.61 | + |
| Freeze dryer room | Equipment surface | 31 | 32.36 | + |
| Bottle washer room | Table | 32 | 27.98 | + |
|  | Table | 33 | 28.36 | + |
| Rough wash room | Table | 34 | 27.08 | + |
|  | Ground | 35 | 26.51 | + |
| Air outlet of fermentation room | Outdoor air outlet | 36 | 33.67 | + |
|  | Outdoor air outlet | 37 | None | - |
|  | Ground | 38 | None | - |
| Air outlet of sub-packing workshop | Outdoor air outlet | 39 | 33.79 | + |
|  | Outdoor air outlet | 40 | 35.65 | + |
| Air outlet of freeze dryer room | Outdoor air outlet | 41 | None | - |
|  | Outdoor air outlet | 42 | None | - |
| Fermentation workshop | Return air inlet | 43 | None | - |
|  | Return air inlet | 44 | None | - |
| Sub-packing workshop | Return air inlet | 45 | 30.88 | + |
|  | Return air inlet | 46 | 28.55 | + |
| Visitors gallery | Emergency exit | 47 | 29.6 | + |
| Product warehouse | Vaccine vials | 48 | 20.19 | + |
|  | Vaccine vials | 49 | 23.58 | + |
|  | Vaccine vials | 50 | 23.89 | + |
|  | Vaccine vials | 51 | 22.79 | + |
|  | Vaccine vials | 52 | 27.52 | + |
|  | Vaccine vials | 53 | 22.49 | + |
|  | Vaccine vials | 54 | 25.26 | + |
|  | Vaccine vials | 55 | 25.65 | + |
|  | Vaccine vials | 56 | 22.50 | + |
| Quality inspection center | Vacuum drying oven | 57 | 34.96 | + |
|  | Vacuum drying oven | 58 | 21.38 | + |
|  | Analytical balance | 59 | 29.84 | + |
| Sterile room (a) | Biosafety cabinet | 60 | 33.75 | + |
|  | Incubator | 61 | 32.37 | + |
|  | Ground | 62 | 30.65 | + |
|  | Table | 63 | 31.87 | + |
| Sterile room (b) | Buffer room | 64 | 28.64 | + |
| Sterile room (c) | Incubator | 65 | 32.87 | + |
| Nucleic acid extraction room | Biosafety cabinet | 66 | 31.82 | + |
|  | Centrifuge | 67 | 24.47 | + |
| Outside the workshop | Tree | 68 | 28.06 | + |
|  | Telegraph pole | 69 | 36.41 | + |
|  | Green plant | 70 | None | - |
| Outside workshop environment | Ground | 71 | 39.27 | Suspected |
|  | Ground | 72 | 39.14 | Suspected |
|  | Administrative office doorknob | 73 | None | - |
|  | Conference room table | 74 | None | - |
|  | Administrative office wall | 75 | None | - |
|  | Quality control center doorknob | 76 | None | - |
|  | Rockery | 77 | 38.08 | Suspected |
|  | Factory exterior wall | 78 | None | - |
|  | Administrative building bathroom | 79 | None | - |
| Negative control | NA | NA | None | Valid |
| Positive control | NA | NA | 14.63 | Valid |

RT-PCR positive: Ct value ≤38

RT-PCR negative: no amplification curve

Suspected: 38＜Ct value ≤40
